# Association of hyperuricemia and serum uric acid lowering therapy with mortality in hemodialysis patients

**DOI:** 10.1080/0886022X.2020.1835674

**Published:** 2020-10-19

**Authors:** Benjamin Rohn, Wiebke Jansing, Felix S. Seibert, Thiemo Pfab, Okan Cinkilic, Jürgen Paßfall, Sven Schmidt, Nina Babel, Frederic Bauer, Timm H. Westhoff

**Affiliations:** a Medical Department I, Universitätsklinikum Marien Hospital Herne, Ruhr-University, Bochum, Germany; b MVZ Diaverum Potsdam, Potsdam, Germany; c Dialysezentrum Schwerte, Schwerte, Germany; d Nierenzentrum Charlottenburg, Berlin, Germany; e Dialysezentrum, Fürstenwalde, Germany

**Keywords:** Chronic kidney disease, hyperuricemia, hemodialysis, mortality, gout

## Abstract

**Introduction:**

In the general population, hyperuricemia is associated with increased morbidity and mortality. Data on this association in hemodialysis patients is controversial. Moreover, it remains elusive whether serum uric acid (SUA) lowering therapy is associated with mortality.

**Methods:**

Retrospective analysis of 601 patients on chronic hemodialysis therapy in five outpatient centers with a maximum follow-up of 100 and a mean follow-up of 41 months. Death was defined as primary endpoint. Cumulative survival was analyzed by Kaplan–Meier analysis and Cox regressions adjusted for age.

**Findings:**

Cumulative survival rates were higher for those subjects with a higher than median SUA concentration both based on mean annual and baseline measurements (*p* < 0.05 each). There was no survival difference anymore after adjustment for age (*p* > 0.05 each). Stratification for SUA lowering therapy (allopurinol/febuxostat) had no impact on cumulative survival, neither in Kaplan Meier nor in Cox regression analyses (*p* > 0.05 each). Furthermore, Cox regression analysis excluded an increased cardiovascular mortality in subjects with hyperuricemia.

**Discussion:**

In contrast to the general population, hyperuricemia is not associated with increased mortality in patients undergoing hemodialysis. Moreover, xanthine oxidase inhibition was not associated with a survival benefit in this analysis. These data do not support the use of SUA lowering medication in hemodialysis patients with asymptomatic hyperuricemia.

## Introduction

In the general population hyperuricemia is an independent marker of adverse cardiovascular outcome and all-cause mortality. It is associated with hypertension, coronary heart disease, heart failure, and the insulin resistance syndrome [1–3]. Subjects with increased serum uric acid (SUA) levels have an increased mortality independent of preexisting cardiovascular disease [4–6]. The association of hyperuricemia and increased cardiovascular risk is extraordinarily consistent among large epidemiologic studies and different healthy and nonhealthy populations [1,4,7,8]. Data on the association of SUA levels and mortality in hemodialysis patients, however, are controversial. Whereas two registry analyses find a survival benefit in hyperuricemic patients undergoing hemodialyis, two other works do not find this association [9–12]. Petreseki et al. describe in two studies the traditional association of hyperuricemia and increased risk of death in the hemodialysis population and in CKD patients who transition to hemodialysis respectively [12,13].

Cardiovascular risk is determined by a complex interplay of numerous traditional and nontraditional risk factors in end-stage renal disease (ESRD). Interestingly, some classical cardiovascular risk factors of the general population cannot be applied to hemodialysis patients. The most prominent example is the ‘reverse epidemiology’ of LDL cholesterol and mortality [14]. Therefore, it is not self-evident that the general population's association between SUA concentrations and cardiovascular risk exists in hemodialysis patients in the same way. Moreover, it remains elusive whether SUA lowering treatment may affect cardiovascular outcome in this population.

The present retrospective study has three primary goals: First, to analyze the association of hyperuricemia and mortality in hemodialysis patients. Second, it investigates whether xanthine oxidase inhibition is associated with improved survival rates. Finally, it aims to assess the risk for gout attacks in hyperuricemic hemodialysis patients.

## Materials and methods

### Study design and participants

We performed a retrospective analysis of 601 patients on chronic hemodialysis therapy. Data was obtained from five dialysis outpatient centers in Germany. A period of ≥ 3 months on maintenance hemodialysis therapy and an age ≥ 18 years were defined as inclusion criteria. Exclusion criteria were lack of data on SUA concentrations before and/or within three months after initiation of hemodialysis treatment or loss of follow-up due to transfer to another dialysis center. In order to attain a sufficient number of mortality endpoints, data collection was started in several centers in parallel seeking a 2:1 ratio of historical to present patients. Based on our previous analyses we defined an aspired study size of *n* = 600 [15,16]. Recruitment was stopped after enrolling 601 patients. Subjects with a history of gouty arthritis, tophaceous gout, urate nephropathy or uric acid nephrolithiasis were classified as ‘symptomatic hyperuricemia’, otherwise as ‘asymptomatic hyperuricemia’. Data assessment comprised epidemiological information, cause of end-stage renal disease (ESRD), concomitant diseases, and cardiovascular risk factors of the study population. The timepoint ‘three months after first treatment’ was defined as baseline examination for two reasons: First, because of the initial metabolic and hemodynamic changes related to the initiation of hemodialysis treatment. Second, because of incomplete data on SUA concentrations at the first dialysis sessions. Afterwards, SUA concentrations were documented annually.

Death was defined as primary endpoint, gout attacks and cardiovascular mortality as secondary endpoint. Historical patients were followed-up until death, present patients until the date of data assessment. Follow-up ranged from 3 to 100 months, median follow-up was 34 months. Subjects were stratified both according to mean annual SUA concentrations during the overall period and baseline SUA concentrations (SUA < vs. ≥ median of overall population each) and by the presence or absence of SUA lowering medication at baseline.

The study was performed in compliance with the Declaration of Helsinki and has been approved by the local ethics committee (16-5861).

### Statistical analysis

Distribution of numeric data was analyzed by the Kolmogorov–Smirnov test. In case of normal distribution, data are presented as mean ± standard deviation, otherwise as median and interquartile range (IQR). Comparison of normally distributed numeric parameters was performed by two-sided two-sample Student’s t-tests. Comparison of dichotomous categorical parameters was performed by Fisher exact test. Nonparametric tests were used for not normally distributed variables (Mann–Whitney U-test). Cumulative survival was analyzed by Kaplan–Meier curves. Cox’s proportional hazards model was used as univariate analysis to estimate hazard ratios (HR) with 95% confidence intervals (CI) for the primary endpoint. Variables with a *p*-value lower than 0.1 were included in the multivariate Cox proportional hazards regression model. *p* < 0.05 was regarded significant. All statistical analyses were performed using IBM SPSS Statistics, Version 20.0 (IBM corp., Chicago, Illinois, USA).

## Results

### Study population

Data from 601 subjects were included in the study according to the above-mentioned criteria. Patients were followed up for up to 100 months with a median follow-up of 34 months (minimum 3 months – maximum 100 months). Mean age of the population was 69 years. The most frequent cause of end-stage renal disease (ESRD) was diabetes mellitus. Nine patients had type-1 diabetes mellitus; the remaining patients had type-2 diabetes mellitus. Cardiovascular comorbidity was high with 92% hypertension, 50% diabetes, and 51% coronary artery disease. The mean of the annual SUA concentrations of each subject (6.3 ± 1.4 mg/dl) was used for further analysis. The median of these mean SUA concentrations was 6.4 mg/dl. This median is very close to the most widely used definition of hyperuricemia (6.5 mg/dl). Thus, 46% of the study population were regarded hyperuricemic. 31% received SUA lowering medication starting any time after the initiation of dialysis. 99% of the study population received Allopurinol and only 1% obtained Febuxostat.

Stratification of the study population by the median SUA concentration over the complete follow-up period were homogeneous for BMI, blood pressure, lipid status, HbA1c, type of diabetes mellitus, renal and cardiovascular comorbidities except for peripheral artery occlusive disease. Moreover, they differed in age, gender, albumin and glomerulonephritis as primary renal disease. (*p* < 0.05 Table 1). The median of the baseline SUA concentrations was 6.4 mg/dl as well. Stratification by baseline concentrations led to two homogeneous groups as well (*p* > 0.05 each, data not shown).

**Table 1.** Baseline characteristics of the Study population.

	Total (*n* = 601)	Mean SUA < 6.4 mg/dl median (*n* = 301)	Mean SUA ≥ 6.4 mg/dl median (*n* = 300)	*p* Value
Female	229	131	98	0.006
Male	372	170	202	
Age (years) (IQR)	71 (16)	72 (13)	68 (18)	0.001
Body mass index (kg/m^2^) (IQR)	26 (7)	26 (7)	26.1 (8)	0.078
SUA lowering treatment prior to dialysis (Yes/No)	186	110	76	0.004
Mean SUA concentration (mg/dl) (IQR)	6.4 (1.5)	5.5 (1.2)	7.0 (0.8)	<0.0001
Mean systolic blood pressure (mmHg) (IQR)	134.6 (19.5)	135.3 (21.1)	134 (19)	0.763
Mean diastolic blood pressure (mmHg) (IQR)	70 (11.9)	70 (12.7)	70 (11.1)	0.966
Mean LDL cholesterol (mg/dl) (IQR)	91.3 (44)	92.7 (45)	89.8 (42.5)	0.863
Albumin (g/dl)	3.7 (0.62)	3.5 (0.67)	3.9 (0.52)	<0.0001
Angiotensin converting enzyme inhibitor	206	94	112	0.067
Angiotensin receptor blockers therapy	129	65	64	0.940
Losartan therapy	7	5	2	0.271
Loop diuretic therapy	460	224	236	0.219
Atorvastatin therapy	17	7	10	0.45
Alpha adrenergic antagonist therapy	76	37	39	0.721
Calcium Channel Blockers therapy	290	148	142	0.865
Median Standl Biermann Score (IQR)	2 (2)	1 (2)	2 (3)	0.068
HbA1c (%) (IQR)	5.8 (1.3)	5.8 (1.3)	5.9 (1.4)	0.687
Primary renal disease				
Glomerulonephritis	97	34	63	0.001
Interstitial nephritis	34	16	18	0.728
Analgetic-induced nephropathy	6	5	1	0.216
Cystic kidney disease	39	22	17	0.508
Postrenal kidney injury	34	12	22	0.8
Nephrosclerosis	128	75	53	0.036
Diabetes mellitus	200	99	101	0.863
Type I diabetes mellitus	9	5	4	0.710
Type II diabetes mellitus	191	94	97	0.710
Amyloidosis	4	3	1	0.624
Cardiorenal Syndrom	30	21	9	0.38
Unknown	18	8	10	0.642
Other	107	52	55	0.696
Concomitant diseases				
Hypertension (Yes/No)	552/49	275/26	277/23	0.766
Diabetes mellitus (Yes/No)	299/302	152/149	147/153	0.744
Type I diabetes mellitus (Yes/No)	9/592	5/296	4/296	0.741
Type II diabetes mellitus (Yes/No)	290/311	147/154	143/157	0.774
Coronary heart disease (Yes/No)	304/297	152/149	152/148	1.0
Hyperlipidemia (Yes/No)	371/230	179/122	192/108	0.275
Stroke (Yes/No)	104/497	56/245	48/252	0.451
Intermittent claudication (Yes/No)	179/422	74/227	105/195	0.006

Epidemiological information, cause of end-stage renal disease (ESRD), concomitant diseases, and cardiovascular risk factors of the study population. Hyperuricemia – elevated SUA concentrations at any time and/or SUA lowering therapy at any time. Numeric data are presented as mean and standard deviation. Numeric data were tested for statistically significant differences by unpaired t-tests. Categorical data (gender, SUA lowering treatment prior to dialysis, concomitant diseases) were compared by Fisher’s exact test. *p* < 0.05 was regarded statistically significant.

### Association of SUA concentration and mortality

**Figure 1.**
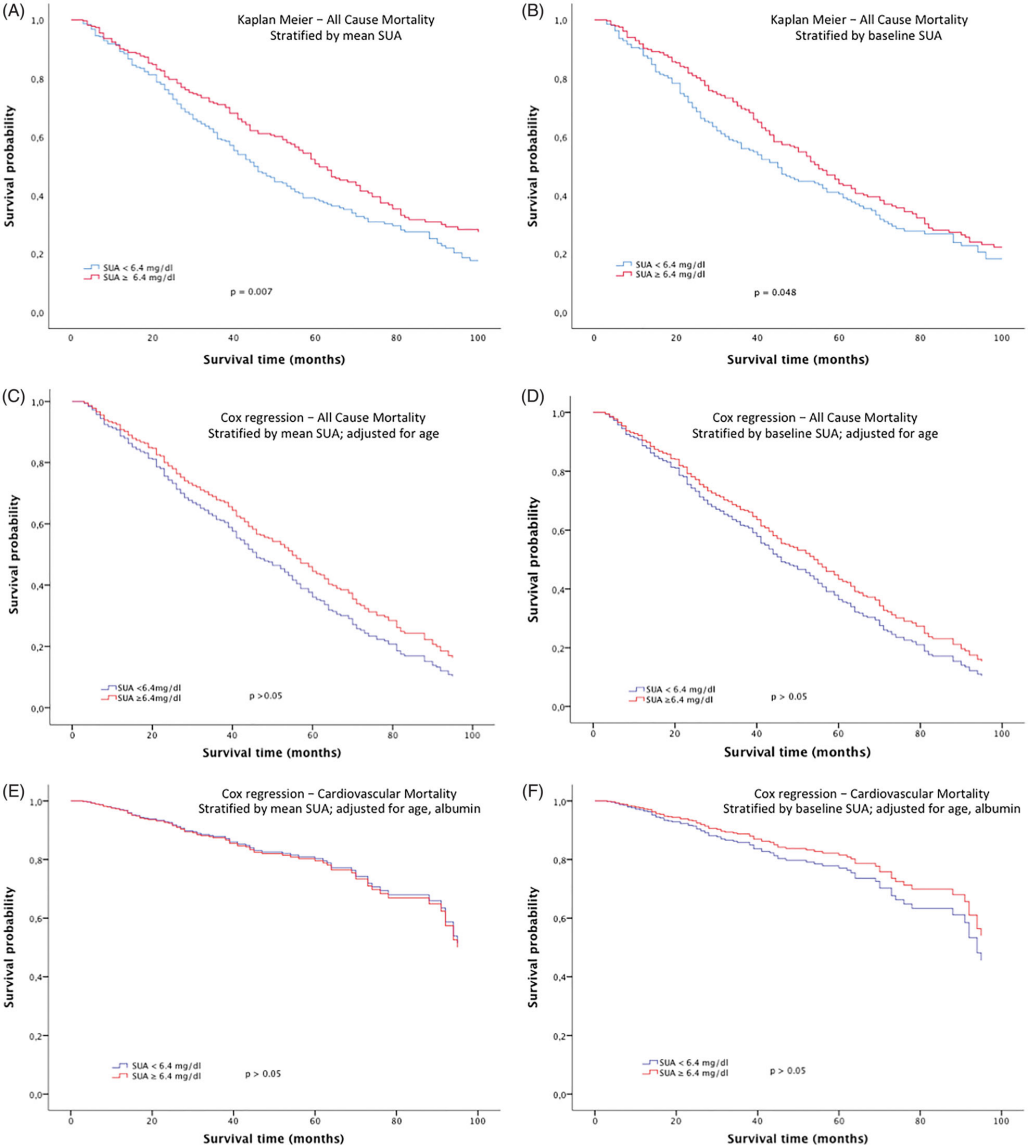
Kaplan–Meier survival curves in dependence of (A) mean SUA concentrations over the complete follow-up period and (B) baseline SUA concentrations (3 months after initiation of dialysis). Cox regression analysis survival curves for all-cause mortality adjusted for age in dependence of (C) mean SUA concentrations over the complete follow-up period, (D) baseline SUA concentrations (3 months after initiation of dialysis). Cox regression analysis for cardiovascular mortality adjusted for age and albumin in dependence of (E) mean SUA concentration and (F) baseline SUA concentration.

In a first approach, the association of SUA concentrations and mortality was analyzed. Kaplan Meier curves demonstrated a higher cumulative survival for both those subjects with a higher than mean SUA concentration over the overall follow-up (Figure 1(A)) and a higher than median SUA concentration at baseline (Figure 1(B)). Univariate Cox regression analysis showed a significant association for age (*p* < 0.0001), but not for gender, BMI, and albumin. Therefore, age was included in the multivariate Cox regression survival analysis (Table 2). After adjustment there was no significant difference in cumulative survival anymore, neither in dependence of mean SUA concentrations over the complete follow up (Figure 1(C)) nor in a higher than median SUA concentration at baseline (Figure 1(D)). Moreover, Cox regression analyses for cardiovascular mortality did not show any difference for subjects with and without hyperuricemia (Figure 1(E,F)).

**Table 2.** Univariate and multivariate Cox porportional hazards regression analyses for mortality.

All Cause Mortality	Univariate Analysis		Multivariate Analysis (A)		Multivariate Analysis (B)	
Risk factor	*p*-Value	HR(95%CI)	*p*-Value	HR(95%CI)	*p*-Value	HR(95%CI)
SUA baseline	0.051	1.246 (0.999–1.554)	0.714	1.060 (0.777–1.446)		
Mean SUA	*0.009*	1.190 (0.956–1.481)			*0.577*	1.095 (0.795–1.509)
Age	*<0.0001*	1.055 (1.044–1.067)	*<0.0001*	1.050 (1.035–1.065)	*<0.0001*	1.050 (1.035–1.066)
Gender	0.411	0.912 (0.732–1.136)				
BMI	0.199	0.987 (0.967–1.007)				
Albumin	0.430	0.949 (0.832–1.081)				
Standl /Biermann Score	*0.122*	0.940 (0.869–1.017				
HbA1c	*0.691*	1.058 (0.8–1.4)				
Blood Glucose	*0.332*	1.003 (0.997–1.010)				
LDL Cholesterol	*0.002*	0.986 (0.977–0.995)	0.627	0.998 (0.990-1.006)	0.607	0.998 (0.990–1.006)
HDL Cholesterol	*0.251*	1.010 (0.993–1.027)				
Total Cholesterol	*0.014*	0.001 (0.984–0.998)	0.536	0.998 (0.991-1.005)	0.566	0.998 (0.991–1.005)
Angiotensin converting enzyme inhibitor therapy	*0.939*	0.991 (0.792–1.241)				
Angiotensin receptor blockers therapy	*0.208*	1.182 (0.911–1.532)				
Losartan therapy	*0.907*	1.07 (0.343–3.337)				
Loop diuretic therapy	*0.206*	0.848 (0.657–1.095)				

Univariate and multivariate Cox proportional hazard regression analyses using all cause mortality as dependent and age, gender, body mass index (BMI), serum albumin, baseline (A) or mean (B) serum uric acid (SUA), Standl Biermann Score, HbA1c, blood glucose, LDL-Cholesterol, HDL- Cholesterol and Total Cholesterol, Angiotensin converting enzyme inhibitor therapy, Angiotensin receptor blockers therapy, Losartan therapy, Loop diuretic therapy as independent variables. *p* < 0.05 was regarded statistically significant.

### Association of SUA lowering medication and mortality

**Figure 2.**
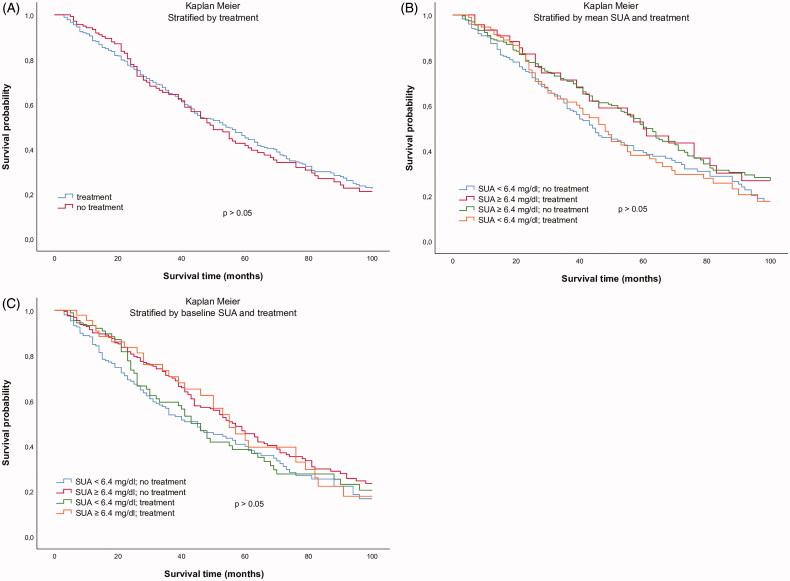
Kaplan–Meier survival curves in dependence of (A) presence of SUA lowering therapy at baseline, (B) mean SUA concentrations and the presence of SUA-lowering therapy at baseline, and (C) baseline SUA concentrations and the presence of SUA lowering therapy at baseline (3 months after initiation of dialysis).

**Figure 3.**
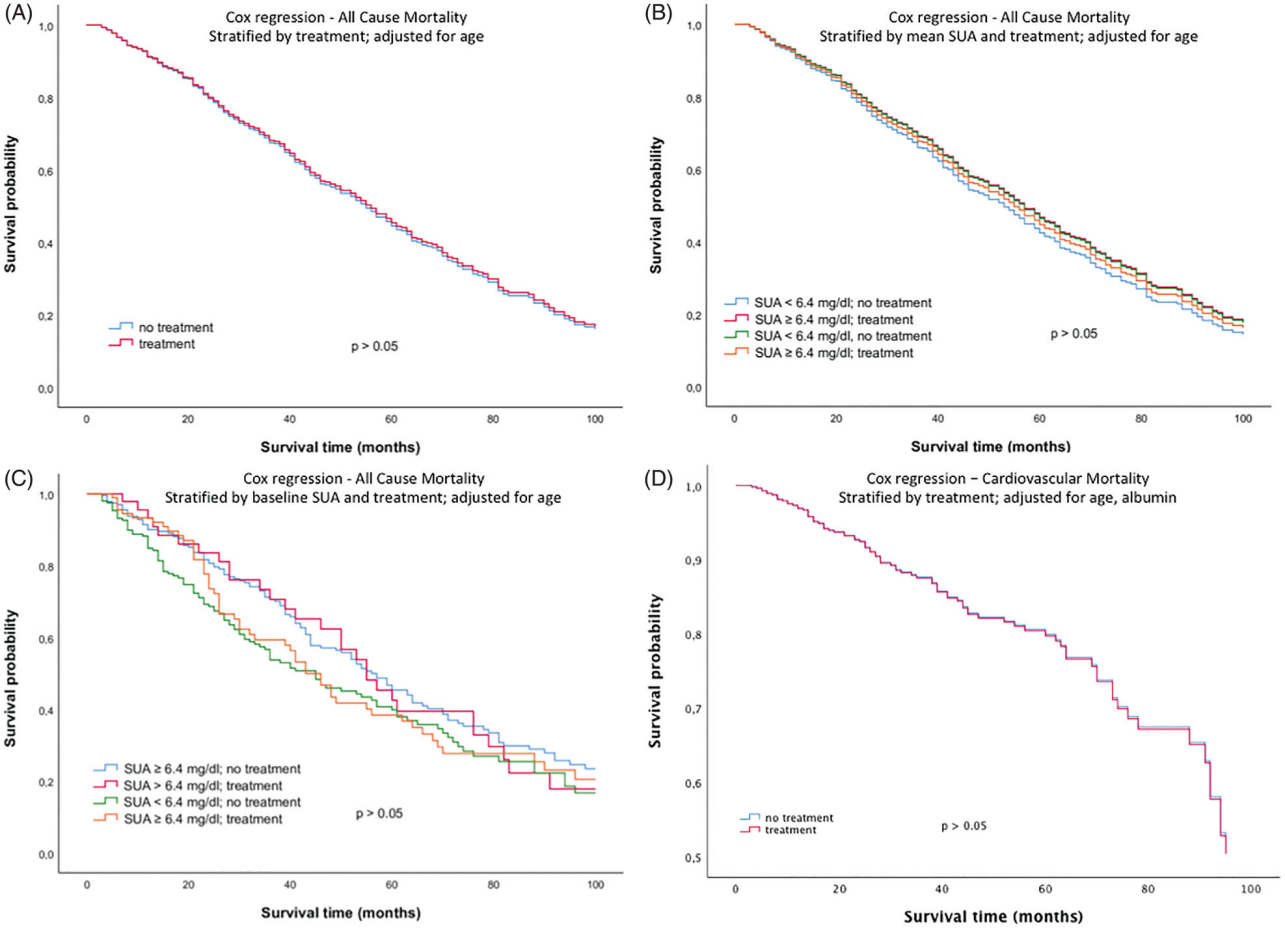
Cox regression analysis survival curves for all-cause mortality adjusted for age in dependence of (A) the presence of SUA lowering therapy at baseline, (B) mean SUA concentrations and the presence of SUA lowering therapy at baseline, (C) baseline SUA concentrations and the presence of SUA lowering therapy at baseline (3 months after initiation of dialysis) and (D) Cox regression analysis survival curves for cardiovascular mortality adjusted for age and albumin in presence of SUA lowering therapy.

The second approach investigated a potential association of SUA lowering medication with mortality. There was no difference in cumulative survival between subjects with and without SUA lowering medication (exclusively xanthine oxidase inhibition) at baseline (Figure 2(A)). Moreover, there was no significant difference after stratification of the study population into four groups based on both SUA lowering therapy and SUA levels (Figure 2(B/C)). Cox regression analysis survival curves adjusted for age in dependence of SUA lowering therapy at baseline did not show any significant difference either (*p* > 0.05, Figure 3(A)). Additional inclusion of SUA concentrations in the multivariate Cox regression model did not show any difference in cumulative survival either (Figure 3(B/C)). Moreover, with regard to cardiovascular mortality, Cox regression analysis adjusted for age did not show any difference in subjects with and without SUA lowering therapy (Figure 3(D)).

### Occurrence of gout attacks in dependence of SUA lowering therapy

In a final step, the occurrence of gout attacks after initiation of dialysis was analyzed. Before starting hemodialysis, 46% of those subjects with SUA lowering medication at baseline had a history of gouty arthritis or tophaceous gout. Thus, 54% of the population received SUA lowering medication for asymptomatic hyperuricemia. Among the overall study population, only 3.6% developed one or more gout attacks during the follow up period. Among those subjects with a mean SUA concentration ≥ 6.4 mg/dl over the complete follow-up period 4.0% developed one or more gout flares, among those with <6.4 mg/dl 3.4%. Thus, the hyperuricemia-associated risk for a gout attack was 1.17 compared to subjects without hyperuricemia. Only 10% of the subjects with prior symptomatic hyperuricemia developed gout attacks after the initiation of hemodialysis, all of them despite medication.

## Discussion

Hyperuricemia is associated with increased cardiovascular morbidity and mortality in the general population [5,15,17,18]. The present data show, that this association does not exist in the same way for patients on hemodialysis. It is unknown, whether the association of hyperuricemia and cardiovascular mortality in the general population has a causal character or not. Hyperuricemia is frequently associated with other cardiovascular risk factors like adipositas, hyperlipidemia, insulin resistance and hypertension. Hence, hyperuricemia could simply constitute a surrogate marker for these parameters. On the other hand several small studies demonstrated a blood pressure lowering effect of the xanthine oxidase inhibitors allopurinol and febuxostat [19,20]. Moreover, allopurinol improved endothelial function and local blood flow in hyperuricemic subjects with congestive heart failure and improved ventricular function in animal models [21,22].

In the present hemodialysis population, Kaplan–Meier analyses show no advantage of normouricemia on cumulative survival at all. A multitude of reasons can be envisioned to explain this finding. E. g., SUA concentrations are not only determined by genetic predisposition, body weight, renal function and dietary habits in ESRD, but also depend on elimination by dialysis. Moreover, the pathophysiology of arteriosclerosis and atherosclerosis is much more complex in ESRD. It encompasses mechanisms that do not play the same role in the general population, such as the differentiation of vascular smooth muscle cells to osteoblast like cells [23].

But why do hyperuricemic subjects counterintuitively show a lower mortality than normouricemic ones? This finding resembles the ‘reverse epidemiology phenomenon’ of LDL cholesterol and mortality in hemodialysis patients. Apparently, not all well-established associations of traditional cardiovascular risk factors and outcome can simply be applied to the ESRD population. The increasing mortality of hemodialysis patients with very low LDL cholesterol concentrations is supposed to be a consequence of malnutrition. BMI had no impact on survival, however, in the present population.

The Cox regression analysis shows that the apparent reverse epidemiology phenomenon has to be explained by differences in age in normo- and hyperuricemic subjects. Subjects with hyperuricemia were younger than those with hypouricemia. Interestingly, stratification approaches based on SUA concentrations over the complete follow-up versus a single SUA assessment after the initiation of dialysis yielded consistent findings. Thus, an increased SUA concentration after the initiation of dialysis does not seem to necessitate the initiation of SUA lowering therapy from a cardiovascular point of view. In agreement, SUA lowering therapy (allopurinol or febuxostat) revealed no association with cumulative survival, neither in Kaplan–Meier nor in Cox regression analysis. Moreover, Cox regression analysis for cardiovascular mortality showed analogous findings.

Beyond potential cardiovascular benefits, small prospective studies indicated a GFR-sparing effect of allopurinol in chronic kidney disease [24]. The impact of xanthine oxidase inhibition on the progression of chronic kidney disease has been examined in the past [25]. Whereas meta-analysis data from small studies indicated a potential protective effect, the two recently completed large randomized controlled trials PERL and CKD-FIX did not show GFR sparing effects [26–29]. Cardiovascular events and mortality were described as secondary endpoints and were not significantly affected.

The only approved indication for xanthine oxidase inhibition is neither avoidance of cardiovascular events nor nephroprotection. It is the treatment of symptomatic hyperuricemia. In the present hemodialysis population, the incidence of gout attacks was extremely low. This phenomenon is well defined for other inflammatory systemic diseases and attributed to the immune dysfunction in uremia [30]. Indeed, only 3.6% of all hyperuricemic subjects developed a gout attack during the follow-up. Hyperuricemia increased the risk of a gout attack only marginally by 17%.

The findings of the present study are in line with registry data published by Latif and Sugano with regard to the association of higher SUA levels and lower all cause mortality. They are in contrast, however, to smaller studies by Kim and Petreseki [11,12]. Our study adds novel aspects to the existing literature for the following reasons: Sugano et al. describe only one-year mortality and made use of SUA concentrations measured at different time points of dialysis duration. We provide data on an observation period of 3 − 100 months. Moreover, the present findings are based on two analyses using both mean SUA levels over the complete observation period and baseline SUA levels at a predefined time point three months after the initiation of dialysis. Furthermore, the study by Latif did not investigate effects of SUA lowering therapy on gout attacks. Finally, the Cox regression analyses provide a possible reason for the ‘reverse epidemiology’ phenomenon on hyperuricemia and mortality by the adjustment for age.

Many subjects receive SUA lowering medication in the predialysis period. Some of them – in line with current guidelines – because of prior episodes of gouty arthritis or nephrolithiasis, some others because of asymptomatic hyperuricemia. Frequently, xanthine oxidase inhibitor treatment is continued when regular hemodialysis treatment is initiated. Our findings indicate that SUA lowering therapy might be dispensable in hemodialysis patients with asymptomatic hyperuricemia.

The study is limited by its retrospective character. Retrospective analyses are able to detect an association, but they are not able to prove causality. With regard to the generic availability of allopurinol and the short remaining period of the febuxostat patent, it appears very improbable that there will be funding for a randomized trial on the effects of SUA lowering medication on cardiovascular outcome. SUA is influenced by several drugs, for example, Losartan and loop diuretics. We demonstrated that administration of the most important drugs was homogeneous in the two groups, but naturally cannot finally exclude a minor impact of other drugs on SUA concentrations.

In summary, the present analysis shows that – in contrast to the general population – hyperuricemia is not associated with increased mortality in patients undergoing hemodialysis. Moreover, xanthine oxidase inhibition was not associated with a survival benefit in this analysis.
